# Prevalence and determinants of oral health conditions and treatment needs among slum and non-slum urban residents: Evidence from Nigeria

**DOI:** 10.1371/journal.pgph.0000297

**Published:** 2022-04-22

**Authors:** Mary E. Osuh, Gbemisola A. Oke, Richard J. Lilford, Eme Owoaje, Bronwyn Harris, Olalekan John Taiwo, Godwin Yeboah, Taiwo Abiona, Samuel I. Watson, Karla Hemming, Laura Quinn, Yen-Fu Chen

**Affiliations:** 1 Division of Health Sciences, Warwick Medical School, University of Warwick, Coventry, United Kingdom; 2 Faculty of Dentistry, Department of Periodontology and Community Dentistry, College of Medicine, University of Ibadan, Ibadan, Oyo State, Nigeria; 3 Faculty of Public Health, Department of Community Medicine, College of Medicine, University of Ibadan, Ibadan, Oyo State, Nigeria; 4 Institute of Applied Health Research, College of Medical and Dental Sciences, The University of Birmingham, Birmingham, United Kingdom; 5 Faculty of Social Sciences, Department of Geography, University of Ibadan, Ibadan, Oyo State, Nigeria; 6 Warwick Information and Digital Group, University of Warwick, Coventry, United Kingdom; VinUniversity, VIET NAM

## Abstract

Oral diseases constitute a neglected epidemic in Low and Middle-Income Countries (LMICs). An understanding of its distribution and severity in different settings can aid the planning of preventive and therapeutic services. This study assessed the oral health conditions, risk factors, and treatment needs among adult residents in the slum and compared findings with non-slum urban residents in Ibadan, Nigeria. The Multistage sampling was used to select adult (≥18-years) residents from a slum and a non-slum urban sites. Information sought from participants included dietary habits, oral hygiene practices, and the use of dental services. Oral examinations were performed in line with WHO guidelines. Associations were examined using logistic regression. Mediation analysis was undertaken using generalized structural equation modeling. The sample comprised 678 slum and 679 non-slum residents. Median age in slum vs non-slum was 45 (IQR:32–50) versus 38 (IQR:29–50) years. Male: female ratio was 1:2 in both sites. Prevalence of oral diseases (slum vs non-slum sites): dental caries (27% vs 23%), gingival bleeding (75% vs 53%) and periodontal pocket (23% vs 16%). The odds of having dental caries were 21% higher for the slum dwellers compared to non-slum residents (OR = 1.21, 95% CI:0.94 to 1.56); and 50% higher for periodontal pocket (OR = 1.50, 95%CI: 1.13 to 1.98), after adjusting for age and sex. There was little evidence that tooth cleaning frequency mediated the relationship between place of residence and caries (OR = 0.95, 95%CI: 0.87 to 1.03 [indirect effect], 38% mediated) or periodontal pocket (OR = 0.95, 95%CI: 0.86 to 1.04, 15% mediated). Thirty-five percent and 27% of residents in the slum and non-slum sites respectively required the “prompt and urgent” levels of treatment need. Oral diseases prevalence in both settings are high and the prevalence was generally higher in the slum with correspondingly higher levels of prompt and urgent treatment needs. Participants may benefit from targeted therapeutic and health promotion intervention services.

## Introduction

Slum settlements provide homes for about 1 billion world population [1,2]. Slums are characterized by crowded, unhealthy places with a high risk of infection and injury [3,4]. Slum residents are often marginalized and have limited access to basic services [1,5]. Empirical evidence points to higher disease burdens among slum dwellers compared to their rural and urban counterparts [6–9]. This has led many to suggest that slums should be identified and studied separately from other types of urban settings lest the plight of slum dwellers gets lost in urban averages [1,7,10]. The health of slum dwellers has been a major concern to government and researchers as slum dwellers appeared to have comparatively greater health challenges compared to non-slum dwellers within the same urban continuum [6–8]. That said, the determinants of disease between slum and non-slum settings might be less clear-cut when it comes to non-communicable diseases. Whereas there is little doubt that children fare particularly badly in slums as compared to non-slum city areas, the situation for adults is more nuanced. Evidence points to a higher prevalence of smoking [11,12], obesity, and diabetes mellitus [13] among slum dwellers than residents of other parts of the city, although the prevalence of high blood pressure appeared similar in both settings [13].

Oral health is growing in significance as an integral part of health and exerts significant effects on individuals, communities, and the wider society [14–18]. For long, oral health is recognized to be an essential component of overall health and well-being [18–20]. Currently, oral diseases affect an estimated 3.5 billion people globally and untreated dental caries is one of the most prevalent non-communicable diseases [14,21]. The economic burden of oral diseases is considerable [22–24]. From the 2010 Global Burden of Disease Study, the global economic burden of dental diseases amounted to $442 billion yearly, of which $298 billion (4.6% of global health expenditure) was attributed to direct treatment costs and $144 billion to indirect costs in terms of productivity losses due to caries, periodontitis, and tooth loss [22]. We recently carried out a systematic review of population-based surveys of oral health among adult urban residents in LMICs and found only three eligible studies dealing with oral health in slums: one study in Ahmedabad, India compared oral health across slum versus non-slum urban locations [25] and the remaining two from Bangladesh and Indore city, central India examined slum populations exclusively [9,26]. None was found for sub-Saharan African countries. This is in spite of the fact that oral health issues are regarded as a substantial population health burden, especially for the disadvantaged and poor population groups in both the developing and developed countries [27,28]. We, therefore, undertook this study to compare oral health and the determinants of oral health across slum and non-slum city areas. It is worth noting that major risk factors for caries and periodontal disease which include unhealthy sugary foods, tobacco use, excessive alcohol consumption, and poor hygiene practices, are also risk factors for other non-communicable diseases such as cardiovascular diseases, cancer, chronic respiratory disease, and diabetes [14,15,29–31].

In this paper we compared oral health in a slum vs. a non-slum area with the following aims: 1) To find out whether and to what extent differences in oral conditions may occur across slum vs. non-slum areas. 2) To find out if any difference between slum and non-slum areas could simply be attributed to age and gender differences. 3)To examine known determinants (risk factors) of oral diseases by location. 4) To examine the effect of each risk factor on oral conditions net of age, gender, and location. 5) To examine the causal component of risk factors associated with the location identified through the above analyses.

We hypothesized that the determinants of oral health might pull in opposite directions with slum dwellers taking less cariogenic diet than non-slum city inhabitants while the non-slum residents exhibited better dental hygiene and had better access to dental health care [25,32–36].

## Methods

### Design

The study was a descriptive cross-sectional survey with an analytical comparison between slum and non-slum sites in Ibadan, the capital city of Oyo State, South-Western Nigeria. A purposive sampling method was deployed in the selection of the slum (Idikan) and the non-slum urban (Okeado) sites. The sites were selected based on similar ethnic and religious profiles.

### Ethics

Ethical approvals for the research protocol were obtained from the Biomedical and Scientific Research Ethics Committee (BSREC: 37/18-19) of the University of Warwick and the Oyo State Research Ethics Review Committee (AD 13/479/1247) of Ibadan, Nigeria (S1 and S2 Files).

### Sample size calculation

Sample size calculations were made to ensure sufficient statistical power for assessing the prevalence of oral diseases and for comparing the proportions of oral disease between slum and non-slum sites [37,38]. The estimates of prevalence for oral disease outcomes used were 12.8% for dental caries and 75.4% for periodontal disease [39,40]. For estimating oral disease prevalence, the level of confidence was set at 95% and the desired level of precision was 5 percentage points. For comparison between slum and non-slum sites, a difference of 10% was assumed with an alpha of 0.05 and 80% power. Based on the largest sample size suggested by the calculations, a minimum sample size of 650 people in each slum and non-slum setting and a total of 1,300 was required.

### Mapping of study sites for easy sampling

The boundaries of each site were determined together with the National Population Commission (NPopC) staff (a commission vested with the duty of publishing information or data on population for various purposes in Nigeria) who were familiar with the communities. The site boundaries were identified on Google Map from where structures were delineated and digitized using high-resolution satellite imagery from the DigitalGlobe. A building-footprint database was created for each site using this high-resolution satellite imagery. A total of 1,923 and 3711 structures were extracted from the slum and the non-slum areas, respectively.

### Sampling strategy

We used a multistage sampling technique. First, we selected 1000 building structures each for slum and non-slum sites from the structures identified from satellite imagery described above using the random selection algorithm in Quantum GIS software. Second, we located the buildings using the Geographic Positioning System (GPS) technology aided by the local internet network. Teams of researchers created an inventory of all households within each selected building, then selected one household per building at random using the OpenDataKit (ODK) client application. Lastly, we obtained an inventory of all adults in each selected household from which we selected one eligible adult participant at random, again using ODK software.

### Inclusion and exclusion criteria

To be eligible, participants had to be at least 18 years of age and must be residents in the area for at least one year. Visitors, guests, house staff, and chronically ill patients, were excluded from the study.

### Data collection exercise

Following the random selection of an eligible household member to be included in the study, a data collection team visited the selected household to meet the selected person. If the selected adult was available, he/she was enrolled in the survey and when not available, a second and up to a third visit (if needed) was made. During the meeting, the number of visits before a successful meeting was noted, then detailed information about the project was shared, and clarifications were provided, where needed. The participant was asked about their willingness to participate in the research and if yes, they were invited to sign the consent form. After obtaining the participant’s signature, a good location with adequate light and sitting arrangement was identified in the home environment with the assistance of the participant. The participant was then seated on a regular chair and the questionnaire was administered by the trained recording clerk while the dentist prepared for the oral examination (light source—headlamp, fresh tray of sterile and sealed set of examination instruments, wooden spatula, sterile gloves, and face mask). After the administration of the questionnaire, oral examination was performed by the dentist, and findings were entered into the prepared tablets by the recording clerk. Following the oral examination, if a participant was found to have dental problems that required urgent attention, they were referred to the University College Hospital with a unique identifier that entitled them to subsidized cost of care. Others who did not require urgent dental treatment benefitted from oral hygiene practice reinforcement and education.

On the few occasions (8 times) that the participants declined to partake in the research exercise, the reasons for declining were noted and the process was discontinued. Thereafter, another eligible member within the same household was randomly selected as a replacement for the person that declined.

At the end of the data collection exercise, each participant received a tube of toothpaste and a toothbrush as a token of appreciation for their time in participation in the study.

### Instrument validation

The questionnaire for this study was an adaptation of a questionnaire from the National Institute for Health Research—Global Health Research Unit (NIHR-GHRU) [41] and the WHO adult oral health questionnaire [42]. The face validity of the adapted questionnaire was checked by two experts in the field of dental public health.

### Training of data collection teams

Ten teams, each consisting of a qualified dentist and an experienced recording clerk, were trained to collect data. The activities of each team were supervised and monitored by the first author. All team members were trained in understanding and interpretation of the questionnaire as well as the application of criteria and codes for various oral diseases and appropriate recording in line with the WHO standards [42]. The dentists were fresh graduates of the University of Ibadan and were specially trained at the University College Hospital (UCH) dental clinic, to elicit and record findings on oral examination in line with the WHO set criteria [42]. The WHO’s Community Periodontal Index (CPI) probe (a specially designed, lightweight metallic probe) for the assessment of periodontal status and disposable materials [42] were used in the training of the research team members. Examiners’ interrater variability was tested for dental caries, periodontal pocket formation and other oral health conditions, in which 10 examiners interpreted 10 different scenarios for both oral health outcomes.

### Pre-test

A pre-test of the study was conducted at the Abadina community of the University of Ibadan using 50 randomly selected buildings and households. The exercise enabled verification of the veracity of information obtained from satellite imagery by a physical on-the-spot visit of the 50 buildings (5 buildings per team). The exercise also enabled the collection of other useful information on the QField, an Android-based GIS for collecting field data. The processes of sampling study participants using the ODK software were tested. The duration of data collection activities (average time and range; including both questionnaire and oral examination), as well as the recording of information into the tablet computer, were verified. Ambiguity in the questionnaire design was also identified and promptly reviewed.

### Questionnaire

Participants responded to a structured and standardized questionnaire which was interviewer-administered. Information collected included participants’ socio-demographic information, period of residence, the experience of a dental problem within the last 12-month period, self-assessment of oral health status, dental hygiene practices (frequency of tooth cleaning, use of aids for oral hygiene, use of toothpaste containing fluoride), utilization of dental services (dental visits, reasons for dental visits), consumption of sugary foods and drinks, use of tobacco, and consumption of alcohol. The questionnaire was translated into the local (Yoruba) language and back-translated in line with WHO recommendations.

### Oral examination

Oral examinations were performed by the dentist in each team. The WHO’s Community Periodontal Index (CPI) probe and disposable materials [42] were deployed for the oral examination. Mouth examination was carried out with the aid of visual and tactile senses. The teeth were examined systematically from the first to the fourth quadrant. Diagnosis of oral conditions was made in accordance with the WHO criteria for oral health surveys [42] and the findings were recorded using the WHO standardized oral health assessment format which was prepared into the tablet computers. The following information was obtained from all participants: dentition status (carious teeth, missing due to caries and filled teeth for the reason of caries), periodontal status (gingival bleeding and pocket formation); loss of attachment, and intervention urgency [42]. The lifetime caries experience—DMFT (sum of decayed, missing, and filled teeth) [42], was derived for each participant, and the presence and stages of periodontal diseases (gingival bleeding, periodontal pocket, or periodontal attachment loss) were recorded [43]. Sterilization of all clinical dental instruments was done at the end of each day’s exercise at the Primary Oral Health Care Centre, University College Hospital (UCH), Ibadan, based in Idikan.

### Data analysis

Demographic characteristics and oral health outcomes were described by place of residence (slum and non-slum). For categorical variables, counts and percentages were reported. For numeric variables, means and standard deviations or medians and interquartile ranges were reported where appropriate. The frequency of cariogenic food consumption was determined by response to 8 items whose scores were added to construct a continuous variable reporting a sum score for each individual (range 8–48). The mean score was derived and used as a cut-off to produce binary output of less and more frequent intake [44,45]. Alcohol intake within the preceding 30 days was also determined using the mean number of drinks by those who drank as the cut-off to produce a binary output into; moderate and excessive alcohol intake [46].

Our objective was to explore the possible reasons for any differences in oral health outcomes between people living in the slum vs. those in the non-slum area. To this end, we first examined the relationship between place of residence and each of the two main oral health outcomes (dental caries, and periodontal pocket formation).

We chose dental caries and periodontal pocket formation because of their well-known competing risk for tooth loss in the adult population [47–50].

Second, we determined whether particular risk factors such as alcohol use, tobacco use, diet, and oral hygiene habits had a relationship with the place of residence and oral health outcomes. This is because these risk factors independently or collectively play significant roles in the development of oral diseases [51–56]. To establish causal pathways, we predetermined whether certain risk factors (diet and oral hygiene habits) acted as mediators. For example, we explored whether a cariogenic diet mediates the relationship between place of residence and dental caries. These risk factors were chosen as potential mediators for analysis on the basis of our dental knowledge as well as their mediating roles in other situations for example tooth brushing as a mediator between cognitive impairment and oral health outcome [57] and nutrition as a mediator between oral and systemic disease [58].

Associations between oral health outcomes, place of residence, and risk factors were explored using logistic regression models, unadjusted and adjusted for age and sex. Risk factors considered were alcohol intake, tobacco use, cariogenic diet, and frequency of tooth cleaning. Odds ratios, 95% confidence intervals, and p-values were reported. Mediation analysis was performed using generalized structural equation modeling (*gsem* command in Stata). The total effect, direct effect, and indirect effect were calculated using the nonlinear combination command (*nlcom*). The proportion of the relationship mediated was calculated by taking the proportion of the indirect effect divided by the total effect. The effect values from the non-linear combination command were then transformed to odds ratios by taking the exponential. All effects were reported with 95% confidence intervals and p-values. Percentage agreement and Gwet’s AC1 index were used to measure interrater variability of researchers for diagnosing the oral health conditions. The statistical analysis was carried out using IBM SPSS version 26 and Stata version 16.1.

## Results

### Sample characteristics

A total of 1,357 participants were included, evenly distributed between the slum and non-slum residence. Overall, there were 245 (36%) males in slum residences and 234 (35%) males in non-slum residences (Table 1). The average age was higher in the slum residences; the median age was 45 years (interquartile range [IQR]: 32 to 50 years) compared to a median of 38 (IQR: 29–50) in non-slum residences. Oral health perceptions and hygiene practices, and utilization of dental services are summarized in S1 Table. A lower percentage of those in slum residences had seen a dentist (17% versus 24%), used a toothbrush for tooth cleaning (82% versus 98%), and brushed at least twice daily (24% versus 27%) compared to non-slum residents.

**Table 1:** Baseline characteristics by place of residence.

Characteristic	Slum (n = 678)	Non-slum (n = 679)
**Male**	245 (36.1)	234 (34.5)
**Age–median (IQR)**	45 (32 to 60)	38 (29 to 50)
**Age**		
<35 years	192 (28.3)	260 (38.3)
Adult age group (35–44 years)	127 (18.7)	186 (27.4)
45–54 years	114 (16.8)	109 (16.1)
55–64 years	102 (15.0)	61 (9.0)
Elderly age group (65–74 years)	88 (13.0)	40 (5.9)
75 years and above	55 (8.1)	23 (3.4)
**Current marital status**		
Married	437 (64.5)	458 (67.5)
Living together but not married	9 (1.3)	13 (1.9)
Divorced or separated	34 (5.0)	7 (1.0)
Widowed	110 (16.2)	50 (7.4)
Never married and never lived together	88 (13.0)	151 (22.2)
**Highest educational level**		
No education	175 (25.8)	42 (6.2)
Primary school	179 (26.4)	61 (9.0)
Secondary school	285 (42.0)	301 (44.3)
Post-secondary school	32 (4.7)	183 (27.0)
University education	7 (1.0)	92 (13.5)
**Currently working**	533 (78.6)	552 (81.3)
**Length of time of residence in the neighbourhood**		
1–10 years	102 (15.0)	203 (29.9)
11–20 years	103 (15.2)	80 (11.8)
>20years	473 (69.8)	396 (58.3)
**Wealth quintile/status (SES)**		
1st quintile (lowest)	231 (34.1)	40 (5.9)
2nd quintile (lower)	167 (24.6)	105 (15.5)
3rd quintile (middle)	157 (23.2)	114 (16.8)
4th quintile (higher)	97 (14.3)	175 (25.8)
5th quintile (highest)	26 (3.8)	245 (36.1)
**Main drinking water source**		
Piped/ tap water	31 (4.6)	47 (6.9)
Borehole	74 (10.9)	283 (41.7)
Well/ spring/ tanker or cart supply	5 (0.7)	8 (1.2)
Rainwater	64 (9.4)	10 (1.5)
Sachet/ bottled water	504 (74.3)	331 (48.7)

IQR: Inter quartile range; SES: Socio-economic status.

Number and percentages reported unless stated otherwise.

### Oral health conditions

Prevalence of the common oral health conditions among the overall study sample was: dental caries—25%; gingival bleeding—64%; pocket formation—19%; and dental trauma—26%. Other findings include: missing teeth—14%; filled teeth– 2%; attachment loss—10% (S2 Table). Regarding the levels of treatment needed or intervention urgency: very few participants (4%) had no need for dental treatment. Sixty-four percent of the overall study sample required the “preventive or routine” level of dental treatment, while 31% had the “prompt” and “immediate/ urgent” levels of treatment need (S2 Table). Examiners’ assessment for oral health conditions revealed substantial, moderate, and almost perfect levels of interrater agreements for the three common oral health conditions respectively: Dental caries—77% agreement (Gwet’s index: 0.71), periodontal pocket formation—66% agreement (Gwet’s index: 0.49), dental trauma—96% agreement (Gwet’s index: 0.96). Other interrater agreement findings were: filled teeth—94% agreement (Gwet’s index: 0.94); gingival attachment loss- 67% agreement (Gwet’s index: 0.50; enamel fluorosis- 80% agreement (Gwet’s index: 0.74); dental erosion- 86% agreement (Gwet’s index: 0.84).

#### Associations between the place of residence and oral health conditions

**Fig 1:**
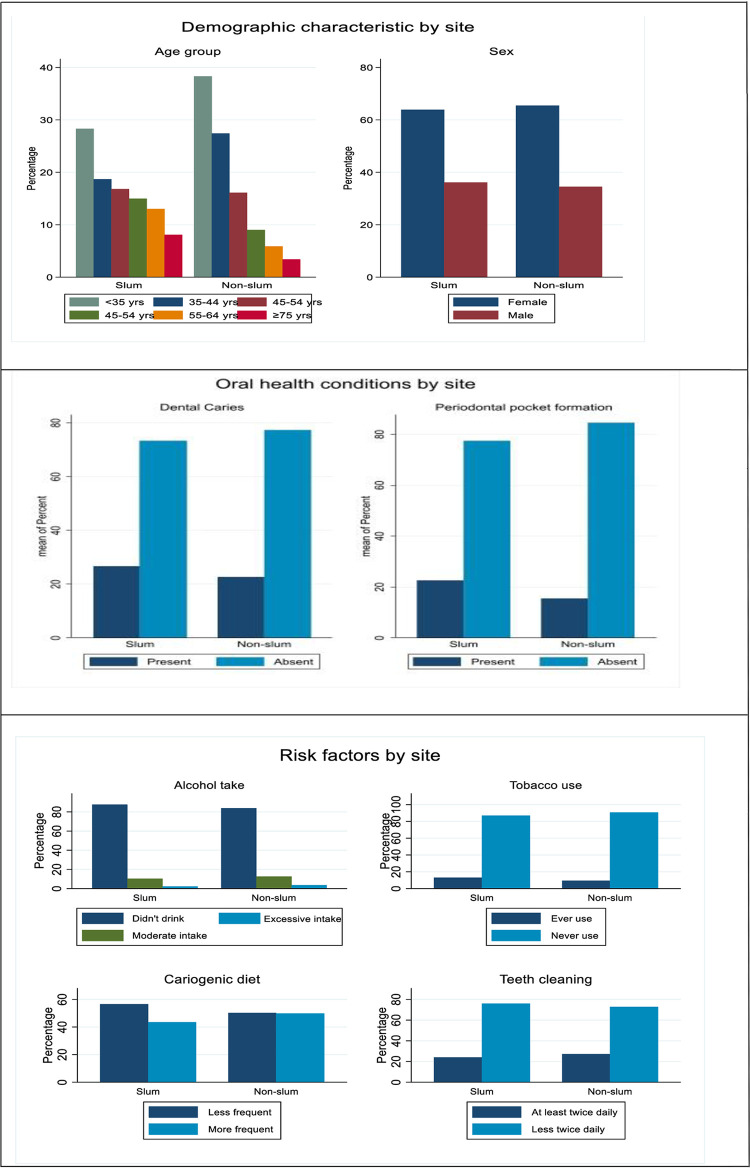
Distribution of key survey data (demographic characteristics, oral health conditions and risk factors) by slum versus non-slum site.

The number and prevalence of oral health conditions among the participants, according to their residential settings, are shown in S2 Table: Dental caries (27% versus 23%) and periodontal pocket formation (23% vs 16%) were more prevalent in slum residences than non-slum residences (Table 2 and Fig 1). Even after adjusting for age and sex, the odds of having dental caries were estimated to be 21% higher for people who lived in slums compared to non-slum residences (OR = 1.21, 95% CI: 0.94 to 1.56); and 50% higher (OR = 1.50, 95% CI: 1.13 to 1.98) for periodontal pocket. The number of participants with no need for dental treatment was 3% and 5% in slum and non-slum sites respectively. The type of treatment required in both sites was mostly preventive/ routine dental treatment and this comprised 62% versus 67% of residents in the slum and the non-slum setting respectively. Prompt and urgent levels of treatment were required for 35% (slum) versus 28% (non-slum) of participants (S2 Table).

**Table 2:** Logistic regression models to explore associations between the place of residence and 1) oral health conditions; 2) risk factors, unadjusted and adjusted for age group and sex.

Characteristic	Slum (n = 678)	Non-slum (n = 679)	Unadjusted odds ratio (95% CI) p-value	Adjusted odds ratio* (95% CI) p-value
**Oral health conditions**				
**Dental caries**	181 (26.7)	154 (22.7)	1.24 (0.97 to 1.59) p = 0.087	1.21 (0.94 to 1.56) p = 0.145
**Periodontal pocket formation**	153 (22.6)	105 (15.5)	1.59 (1.21 to 2.10) p = 0.001	1.50 (1.13 to 1.98) p = 0.005
**Risk factors**				
Alcohol intake**				
Didn’t drink alcohol (last 30 days)	594 (87.6)	570 (84.0)	Reference	Reference
Moderate intake	69 (10.2)	85 (12.5)	0.78 (0.56 to 1.09) 0.147	0.75 (0.52 to 1.08) p = 0.127
Excessive intake	15 (2.2)	24 (3.5)	0.60 (0.31 to 1.15) 0.126	0.68 (0.34 to 1.34) p = 0.265
**Ever used tobacco**	89 (13.1)	63 (9.3)	1.48 (1.05 to 2.08) p = 0.025	1.59 (1.11 to 2.30) p = 0.012
**More frequent cariogenic food consumption**	295 (43.5)	338 (49.8)	0.78 (0.63 to 0.96) p = 0.021	0.90 (0.72 to 1.13) p = 0.365
**Tooth cleaning (at least twice daily)**	163 (24.0)	184 (27.1)	0.85 (0.67 to 1.09) p = 0.197	0.81 (0.63 to 1.04) p = 0.099

* Adjusted age group and sex.

** Multinomial logistic regression used and relative-risk ratios reported.

#### Associations between the place of residence and risk factors for oral health conditions

Alcohol intake for participants was low in both slum and non-slum residence: moderate (10% versus 13%) and excessive (2% versus 4%). Tobacco use was slightly higher in slum compared to non-slum residences (13% versus 9%), whereas higher consumption of cariogenic diet was slightly lower in slum compared to non-slum residences (44% versus 50%). After adjusting for age and sex, the odds of having a higher consumption of cariogenic diet was 10% lower (OR = 0.90, 95% CI:0.72 to 1.13) in slum residences compared to non-slum residences. A slightly lower percentage of participants cleaned their teeth at least twice daily in slum residences compared to non-slum residences (24% versus 27%), After adjusting for age and sex, the odds of tooth cleaning at least twice a day were 19% lower (OR = 0.81, 95% CI: 0.63 to 1.04) in slums compared to non-slum residences (Table 2 and Fig 1).

#### Association between risk factors and oral health conditions

Associations between oral health conditions and potential risk factors, including cariogenic diet, alcohol use, tobacco use, and frequency of tooth cleaning were reported unadjusted and adjusted for age group and sex (S3 and S4 Tables). The odds of having dental caries were 28% higher (OR = 1.28, 95% CI:0.97 to 1.69) for people who cleaned their teeth at least twice daily compared to less than twice daily, adjusting for age and sex. The odds of having periodontal pocket formations was 103% higher (OR = 2.03, 95% CI:0.97 to 4.22) for people who had an excessive intake of alcohol compared to no alcohol intake and 28% higher (OR = 1.28, 95% CI:0.95 to 1.73) for people who cleaned their teeth at least twice daily compared to less, adjusting for age and sex.

#### Validity check for logistic regression

Assumptions of logistic regression were checked for each of the models reported above. All models had binary outcomes; little or no multicollinearity was found between independent variables; the influence of outliers did not affect the results and the observations were independent.

#### Mediation analysis

**Fig 2:**
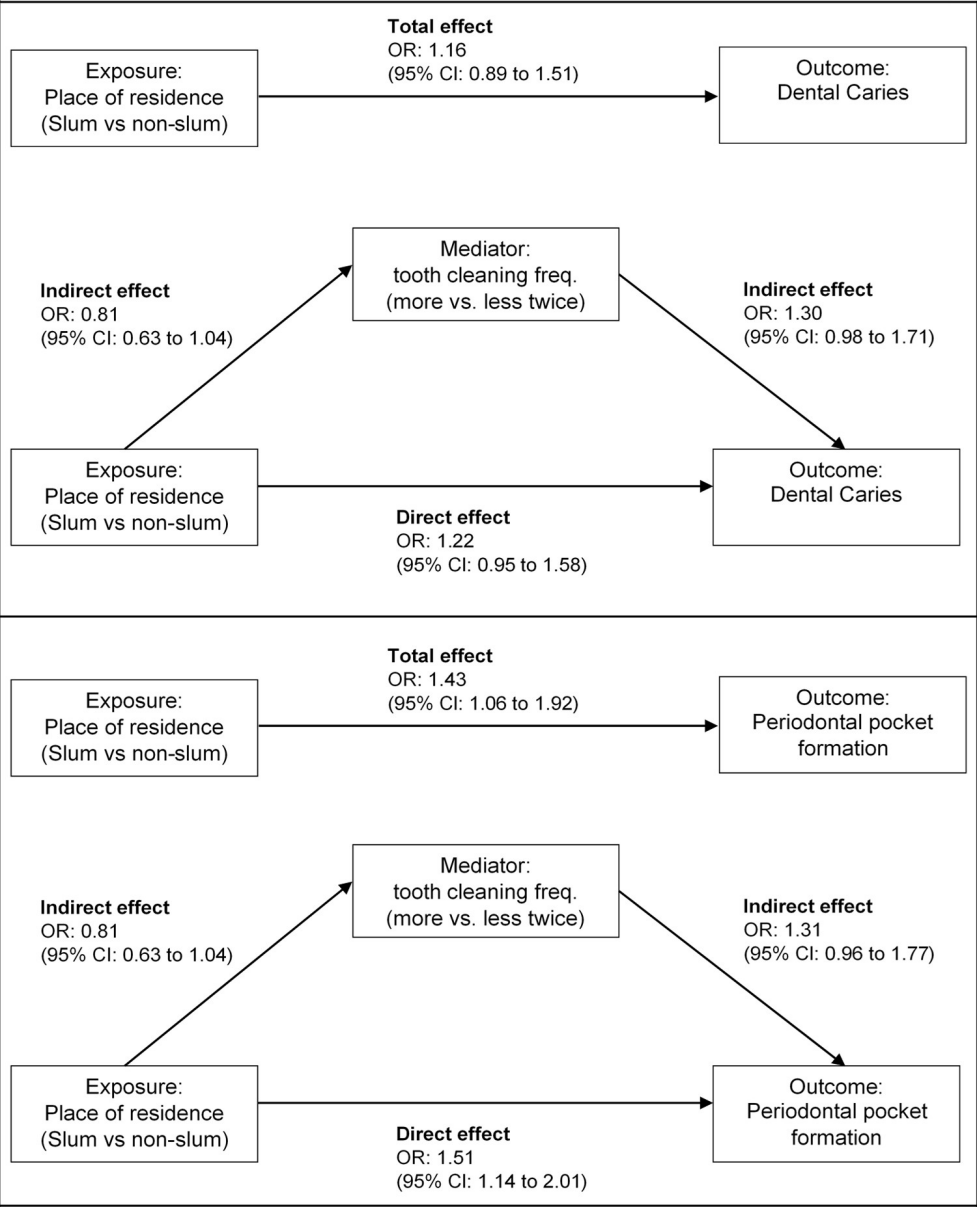
Casual pathways between place of residence and oral health conditions (dental caries and periodontal pocket formation) with frequency of tooth cleaning as a potential mediator, controlling for age group and sex.

*Mediation via tooth cleaning frequency*. The odds of having dental caries were estimated to be 22% higher for people who lived in slums compared to non-slum residents (OR = 1.22, 95% CI:0.95 to 1.58); and 51% higher (OR = 1.51, 95% CI:1.14 to 2.01) for periodontal pocket formation, after adjusting for tooth cleaning frequency, age and sex (direct effects, Fig 2 and Table 3). The odds of tooth cleaning at least twice a day were 19% lower (OR = 0.81, 95% CI:0.63 to 1.04) in slums compared to non-slum residences, adjusting for age and sex (indirect effect). The odds of having dental caries were 30% higher (OR = 1.30, 95% CI:0.98 to 1.71) and periodontal pocket formation was 31% higher (OR = 1.31, 95% CI:0.96 to 1.77) for cleaning teeth at least twice daily compared to less, adjusting for place of residence, age, and sex (indirect effect).

**Table 3:** Generalized structural equation modeling to explore potential mediator frequency of tooth cleaning on the relationship between place of residence and oral health outcomes.

Effect	Odds ratio (95% CI) p-value
**Dental caries as outcome**	
**Direct effect**	
Place of residence → Dental caries	1.22 (0.95 to 1.58) p = 0.123
**Indirect effects**	
Place of residence → Tooth cleaning frequency	0.81 (0.63 to 1.04) p = 0.096
Tooth cleaning frequency → Dental caries	1.30 (0.98 to 1.71) p = 0.067
Total indirect effect	0.95 (0.87 to 1.03) p = 0.224
**Total effect**	
Place of residence → Dental caries	1.16 (0.88 to 1.51) p = 0.288
**Percentage mediated**	38%
**Periodontal pocket formation as outcome**	
**Direct effect**	
Place of residence → Periodontal pocket formation	1.51 (1.14 to 2.01) p = 0.004
**Indirect effects**	
Place of residence → Tooth cleaning frequency	0.81 (0.63 to 1.04) p = 0.096
Tooth cleaning frequency → Periodontal pocket formation	1.31 (0.96 to 1.77) p = 0.085
Total indirect effect	0.95 (0.86 to 1.04) p = 0.225
**Total effect**	
Place of residence → Periodontal pocket formation	1.43 (1.06 to 1.92) p = 0.017
**Percentage mediated**	15%

Analysis adjusted for age group and sex.

*Mediation via cariogenic diet*. The odds of having dental caries were estimated to be 21% higher for people who lived in slums compared to non-slum residents (OR = 1.21, 95% CI:0.94 to 1.56); and 49% higher (OR = 1.49, 95% CI:1.12 to 1.97) for periodontal pocket formation, after adjusting for cariogenic diet, age and sex (direct effects). The odds of having a more frequent cariogenic diet were 10% lower (OR = 0.90, 95% CI:0.72 to 1.13) for people in slum compared to non-slum residents. The odds of having dental caries was 2% higher (OR = 1.02, 95% CI:0.79 to 1.32) and periodontal pocket formation was 41% lower (OR = 0.59, 95% CI:0.44 to 0.78) for having a more frequent cariogenic diet compared to less, adjusting for place of residence, age and sex (indirect effect).

**Fig 3:**
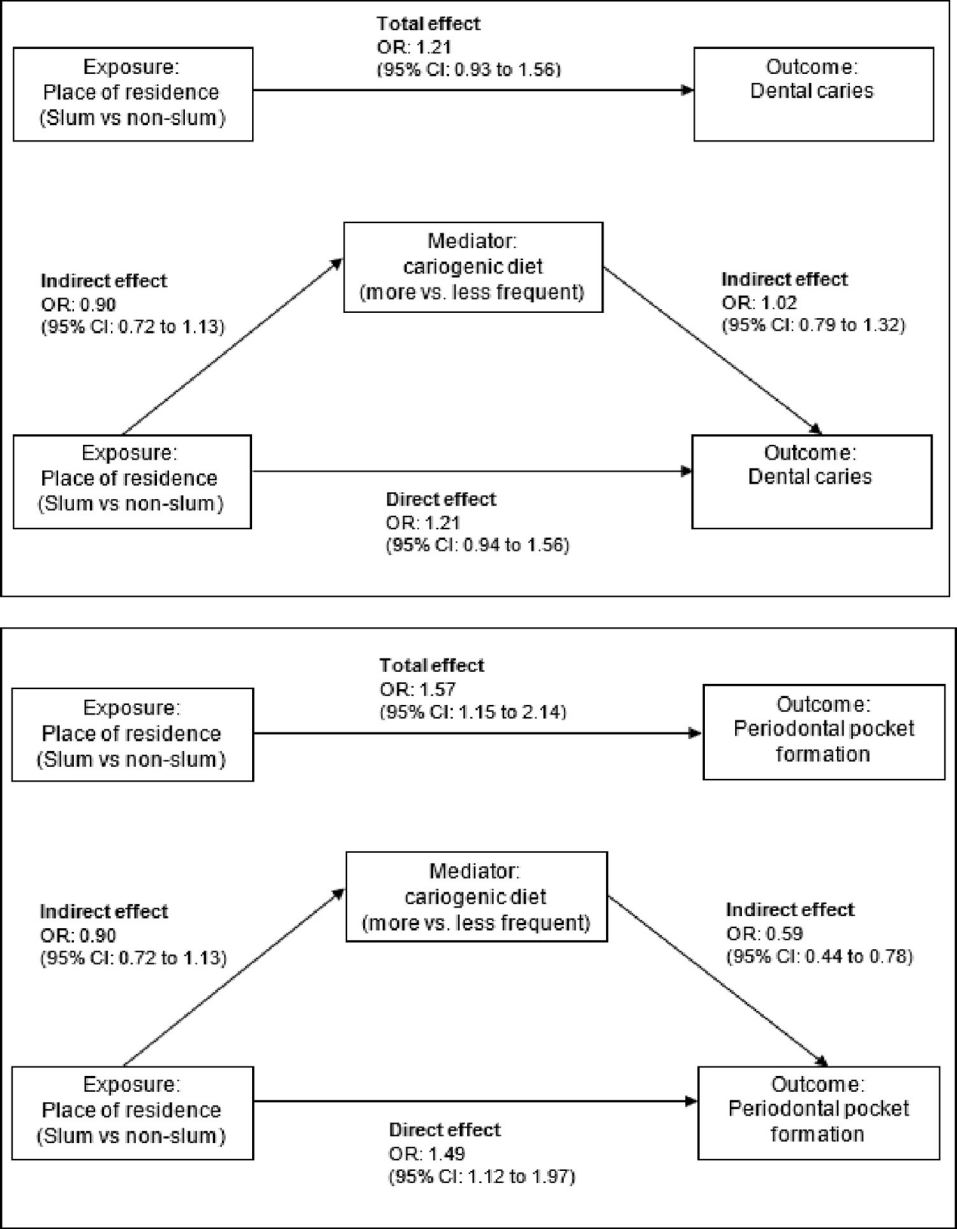
Causal pathways between place of residence and oral health outcomes (dental caries and periodontal pocket formation) with cariogenic diet as a potential mediator, controlling for age group and sex.

There was no evidence cariogenic diet mediated the relationship between place of residence and dental caries (OR = 1.00, 95% CI:0.97 to 1.02 [total indirect effect], estimated 1% mediated) or periodontal pocket formation (OR = 1.06, 95% CI:0.93 to 1.19 [total indirect effect], estimated 12% mediated; see Fig 3 and Table 4).

**Table 4:** Generalized structural equation modeling to explore potential mediator cariogenic diet on the relationship between place of residence and dental outcomes.

Effect	Odds ratio (95% CI) p-value
**Dental caries as outcome**	
**Direct effect**	
Place of residence → Dental caries	1.21 (0.94 to 1.56) p = 0.144
**Indirect effects**	
Place of residence → Cariogenic diet	0.90 (0.72 to 1.13) p = 0.364
Cariogenic diet → Dental caries	1.02 (0.79 to 1.32) p = 0.853
Total indirect effect	1.00 (0.97 to 1.02) p = 0.855
**Total effect**	
Place of residence → Dental caries	1.21 (0.93 to 1.56) p = 0.149
**Percentage mediated**	1%
**Periodontal pocket formation as outcome**	
**Direct effect**	
Place of residence → Periodontal pocket formation	1.49 (1.12 to 1.98) p = 0.006
**Indirect effects**	
Place of residence → Cariogenic diet	0.90 (0.72 to 1.13) p = 0.364
Cariogenic diet → Periodontal pocket formation	0.59 (0.44 to 0.78) p<0.001
Total indirect effect	1.06 (0.93 to 1.19) p = 0.380
**Total effect**	
Place of residence → Periodontal pocket formation	1.57 (1.15 to 2.14) p = 0.004
**Percentage mediated**	12%

Analysis adjusted for age group and sex.

## Discussion

Our study has established that the prevalence of common dental diseases in the two communities studied ranged from moderate to very high for dental caries and periodontal disease respectively. Complete absence of oral diseases was detected in only about 4% of participants who were adjudged to require no form of dental treatment. The high prevalence of some oral diseases such as caries and periodontal disease is comparable to reports from available literature worldwide [59–61]. The fact that these diseases are chronic, silent, and slowly progressive in nature [14–18], often renders them prone to neglect [62,63]. Given the economic effects of dental disease and their effect on general health and well-being [22–24], more attention should be paid to early detection and treatment of dental diseases [23].

Magnifying the already heavy oral disease burden is that the slum residents recorded higher prevalence rates relative to their non-slum counterparts, although the difference was modest. The observed pattern of distribution would seem to mimic the general distribution pattern of non-communicable diseases and risk factors associated with ill-health such as diabetes mellitus, smoking and obesity, which were reported as preponderant in disadvantaged communities in previous studies [6–8].

According to the WHO, dental caries is the most common non-communicable diseases worldwide and is a major public health problem, globally [64]. The global overall prevalence of dental caries for all ages combined as reported in the global burden of disease study 2010 is approximately 35% [65]. In comparison, caries prevalence from our study population is 25%. This lower prevalence may be explained in part by the age of our study participants which is predominantly younger, since the disease is known to increase with age [42]. Other likely determinants of the lower prevalence rates recorded in our study include the habit of consuming less of free sugar [66], fluoride use [67], as well as indigenous caries prevention measures [68]. Further studies to investigate the role and importance of each factor in disease aetiology for our study population should enhance efforts at controlling the same. The difference in the prevalence of caries observed between the slum and the non-slum locations was not significant, even after adjusting for age and gender. However, when the estimated prevalence of dental caries from our study of 27% and 23% in the slum and non-slum urban settings respectively, were compared with that reported from a similar study in India [25], which reported 61% and 47% in the respective settings, the difference was marked. Whereas in our study, participants were selected at random information on how India’s study participants were selected was not recorded and this methodological factor may explain the marked difference in findings between our study and the Indian study.

Access to clean drinking water, especially with respect to its fluoride content, plays a significant role in caries prevention. The main source of fluoride is water, while other sources include food, industrial exposure, drugs, and cosmetics [69]. The WHO, specified a fluoride concentration level of 0.5 mg/L (equivalent to 0.5 parts per million [ppm]) in drinking water for its beneficial effects in the prevention of caries and a tolerance limit of 1.5 mg/L [28,70]. In a tropical environment like Nigeria, a fluoride level of 0.3–0.6 ppm in drinking water was recommended depending on fluoride ingestion from other sources [71]. Although our study did not assess the fluoride concentration in participants’ drinking water, available studies revealed that the majority of drinking water sources (58–75%) in all but one LGA in Nigeria contained 0.3 mg/L or less of fluoride [71]. A recent study conducted in south-west Nigeria reported a smaller proportion (48%) of drinking water with low fluoride from similar sources: ground water- well or borehole [71,72]. Access to clean drinking water is generally a challenge for a large proportion of the Nigerian population but the situation is worse in the slums [73,74]. The non-recognition of slums in official discourses often limit their consideration in the planning of essential public services such as water [74]. The aforementioned may explain why the commonest source of drinking water in both the slum and non-slum areas in our study is packaged water (sachet/ bottle water) followed by borehole. Available reports have confirmed these water sources as clean drinking water sources with adequate fluoride concentration [71,75]. Furthermore, fluoride toothpaste is recommended for the prevention of caries, and is usually advised in conjunction with good oral hygiene [76]. The majority of our study participants reported use of fluoridated toothpaste in cleaning their mouth. Most of the commercially available toothpastes in Nigeria are reported to have the standard recommended concentration of 1000–1500 ppm [72,77,78]. This range is considered both beneficial against caries and safe against dental fluorosis for any age if toothpaste was the individual’s sole source of fluoride. The combination of access to clean/ safe drinking water source and the use of fluoridated toothpaste by the majority of our study participants probably make up for the low fluoride content available in the regions’ ground water. Therefore, there may be justification to shift focus to other causes of caries such as dietary [79], cleaning habits and other risk factors [80].

More than two-thirds of the entire population in our study had periodontal disease. This comprised gingival bleeding of 64%; periodontal pocket formation of 19% and an attachment loss of 10%. Previous studies in different settings, inclusive of slums have utilized various criteria in the assessment of periodontal disease prevalence. These include the Community Periodontal Index of Treatment Needs (CPITN), Clinical Attachment Level (CAL), and/or probing depth (PD). The reported prevalence rates varied but were equally high [25,26,81,82]. Our findings that periodontal disease affected close to three-quarters of the slum dwellers and about half of the non-slum residents further buttresses the heavy burden of periodontal disease being borne by populations in LMICs particularly those residing in slums. However, our findings were again dissimilar to those reported in the study in India, in which the non-slum dwellers had a higher prevalence of gingivitis than slum dwellers while having an equal prevalence for gingival bleeding [25]. This may be explained by their purposively selected small (300) population and that their participants were drawn from across the ages (children and adults). The difference in the prevalence of periodontal pocket formation observed between the slum and the non-slum locations in our study were statistically significant, and remained so, even after adjusting for age and sex. Overall, literature on population oral health surveys among adult residents of LMICs are sparse and more data/studies will be needed to make an adequate comparison.

In terms of the oral healthcare needs, participants in both settings had high needs for oral healthcare, and most of the needs related to preventive/routine treatments. The high treatment need observed in this study is similar to other studies which reported high prevalence of oral diseases [9,26]. However, when the level of treatment required was compared between the two residential locations, more of the slum dwellers required urgent level of care, while more of the non-slum residents required the preventive/routine dental treatments, relative to their counterparts respectively.

In tackling the oral health needs of the populace, access to basic publicly funded oral health services should be central [83,84]. In Nigeria as well as other LMICs, oral health is largely considered less important than the general health, and receives little attention in terms of health care planning and service delivery [85]. Oral health services are delivered in all health care levels (primary, secondary and tertiary) in Nigeria. The primary level of oral care is concerned with the prevention of dental diseases and management of dental emergencies, however, the services are available at only a few primary health care centres (PHCCs) nationwide [79,83,85]. General treatment of oral disorders is provided at the secondary care facilities: private dental clinics, general hospitals, institutions run by faith-based organisations, federal medical centres and armed forces hospitals. Specialised oral health services including treatment of oral diseases and patient rehabilitation are provided at the tertiary (teaching hospitals) [83,85]. Traditional oral health care providers are also available in most regions as well as few non-governmental organisations, all providing oral health services to the people [17,85,86]. The primary level of oral care, is considered as the veritable means to ensure easy access to oral health service to people in all locations, due to its widespread location in the country [83–85], but it has not succeeded in delivering on its service mandate to the people due to persistent challenges (manpower and other resources). Therefore, most oral health services are directed towards the provision of curative and rehabilitative care from the secondary and tertiary levels of care [85]. These levels of care are available mainly in the big cities and largely through out of pocket payment [17]. To date the inclusion of oral health into existing PHCs has continued to suffer setbacks in terms of facilities and capacity building [79,85]. Consequently, a number of independent initiatives targeted at charting a direction for Primary Oral Health Care were set up in Nigeria’s dental schools, but these are too few to meet the current oral health need, they lack adequate manpower and are largely funded from out of pocket [79,85]. The Nigeria’s National Health Insurance Scheme (NHIS) has a mandate, through various prepayment systems, to design and implement a social health insurance scheme that can facilitate easier access to affordable and available quality health care services and achieve a universal health coverage (UHC) [83]. But this equally has its own challenges which range from poor financing of the health system by governments, an absence of prepayment schemes, and a growing population of poorly paid people who reside majorly in slums [83,85]. As such, the NHIS billing system concerning oral health is largely considered only at the level of secondary care [85,87]. These challenges in accessing oral health care may also explain the low use of professional dental services observed among our study participants. A revision of the position of oral health services on the NHIS should advocate to include the coverage of preventive oral health care services as recommended by the WHO [85,88].

Tooth brushing, at least twice daily is the professionally recommended routine that should contribute to individual oral hygiene [89,90]. We found that only a small proportion of our study participants engaged in twice daily mouth cleaning routine across both settings. The indigenous norms, cultural practices, and habits native to the people as well as their limited exposure to correct information from school and through the media may have played a role [68,91]. The low prevalence of mouth cleaning was in stark contrast to findings from a national oral health survey conducted in Malawi which reported a much higher prevalence of 75% for brushing teeth at least twice daily [92]. About 40% in their study reported brushing their teeth three or more times a day. This however, did not seem to reduce the caries prevalence (49%) reported among their adult populations.

In advance of our data analysis, we hypothesized that different determinants would be at play in slum vs. non-slum areas. Previous work had suggested that diets in slums may be less cariogenic than in non-slum urban areas [66,93–95], while at the same time we suspected that hygiene practice and access to dental care may be poorer in slum areas. In the mediation analysis of the association between residential places (slum vs non-slum) and oral health conditions (dental caries and periodontal pocket formation), we found little evidence of either the frequency of tooth cleaning or cariogenic diet being potential mediators. This was unexpected given the established influence of these factors on oral health. The lack of a mediation effect may partly reflect the relatively small observed differences in tooth cleaning practice, cariogenic diet consumption, and dental caries between slum and non-slum populations. Previous studies have linked the pattern of sugar consumption [96–98] and tooth brushing [99,100] with periodontal disease. The unexpected finding in our study may be due to the effect of unknown confounders which require further investigation. They may also indicate issues related to self-reported measures of cariogenic diet consumption and tooth cleaning practice, and the grouping of exposure for these variables. For example, how the respondents’ self-reported brushing frequencies approximate their actual tooth brushing behavior is not clear. In addition, the act of tooth brushing itself is complex as it combines many other variables such as the duration of brushing, the design and quality of the brush, the brushing method and the toothpaste used [99–102]. We have used brushing at least twice daily as the threshold for defining higher versus lower frequency of tooth cleaning, but a previous systematic review suggested the effect might be similar even with brushing at least once daily [99]. Maintenance of good oral hygiene is the foundation on which most oral diseases can be prevented [103]. However, achieving good oral hygiene involves more than brushing the teeth twice daily. A combination of quality in the act of tooth brushing itself [99–102], with the right cleaning frequency, is considered essential and plays a significant role in achieving the level of hygiene that can be effective against oral diseases [89,91]. Moreover, the role of professional tooth cleaning at regular intervals in inhibiting oral diseases should not be overlooked [54,104]. Data from our study suggested that the majority of both the slum and the non-slum residents had never visited a dentist similar to other studies [68,105]. Lastly, our study is cross-sectional and one may indeed require longitudinal studies to properly assess a causal effect. On the whole, our hypotheses regarding key determinants of oral health conditions in slum versus non-slum urban settings were not supported by the data that we observed.

### Study limitations

Our study has certain limitations. First, the study was carried out in only one slum and one non-slum area, and so the findings may not be generalizable to all slums. Second, the cross-sectional design precludes causal inferences, hence only associations can be drawn. However, our study has some major strengths: To the best of our knowledge, it is the first community oral health study in the LMICs to deploy the use of GIS software with GPS technology to select representative samples from randomly selected households and buildings in dense urban areas such as the slums. In the slums, it is rarely feasible to conduct a comprehensive door-to-door survey of every individual or household unit, that can ensure representativeness [41,106]. The adoption of this approach in our study is therefore an innovation among oral health studies.

Additionally, we utilized the latest in the manual series of WHO oral health survey methods [42] in the assessment of public oral health status. The methods were recommended for all oral health surveys to enhance easy comparison of results among populations globally. Lastly, our study is the second and currently, the largest of its kind in the LMICs to compare oral health survey findings related to the prevalence of oral diseases and their determinants among adults residing in the slum and non-slum settings, making a significant contribution to the currently sparse evidence base.

### Conclusion

Oral disease prevalence is high in both the slum and the non-slum urban locations, and the prevalence is generally higher in the slum relative to the non-slum setting. We did not find clear evidence of the mediation effect of cariogenic diet consumption and tooth brushing frequency on the relationship between place of residence and oral health conditions. More than a third and more than a quarter of the slum and the non-slum residents respectively required the prompt and urgent levels of dental treatments. The participants may benefit from interventions targeted at therapeutic, preventive, and oral health promotion services.

## Supporting information

S1 Table Oral health perceptions, hygiene practices, and utilization of dental services by place of residence. (DOCX)

S2 Table Oral health conditions following dental examination. (DOCX)

S3 Table Logistic regression models exploring associations between dental caries and risk factors. (DOCX)

S4 Table Logistic regression models exploring associations between periodontal pocket formation and risk factors. (DOCX)

S1 File Biomedical and Scientific Research Ethics Committee full approval letter. (PDF)

S2 File Oyo state Research Ethics Review Committee full approval letter. (PDF)
