# Supplementary material for: Prevalence and determinants of oral health conditions and treatment needs among slum and non-slum urban residents: Evidence from Nigeria
Source: PLOS Glob Public Health. 2022 Apr 22;2(4):e0000297. doi: 10.1371/journal.pgph.0000297 (PMC10021815; doi:10.1371/journal.pgph.0000297)
Supplement: S1 Table — (DOCX) [file pgph.0000297.s001.docx]

***S1 Table: Oral health perceptions, hygiene practices, and utilization of dental services by place of residence. Number and percentages reported unless stated otherwise***

| **Characteristic** | **Slum (n=678)** | **Non-slum (n=679)** |
| --- | --- | --- |
| **Good self-perception of overall dental health** | 444 (65.5) | 352 (51.9) |
| **Self-reported state of teeth** |  |  |
| Excellent | 68 (10.0) | 88 (13.0) |
| Very good | 130 (19.2) | 205 (30.2) |
| Good | 333 (49.1) | 308 (45.4) |
| Average | 117 (17.3) | 64 (9.4) |
| Poor | 29 (4.3) | 13 (1.9) |
| Very poor | 1 (0.2) | 1 (0.2) |
| **Self-reported state of gums** |  |  |
| Excellent | 72 (10.6) | 102 (15.0) |
| Very good | 122 (18.0) | 203 (29.9) |
| Good | 345 (50.9) | 300 (44.2) |
| Average | 121 (17.9) | 67 (9.9) |
| Poor | 17 (2.5) | 5 (0.7) |
| Very poor | 1 (0.2) | 2 (0.3) |
| **Need for dental care in past 12 months** | 87 (12.8) | 133 (19.6) |
| **Pain/discomfort from teeth/mouth in past 12 months** |  |  |
| No | 188 (27.7) | 216 (31.8) |
| Yes | 471 (69.5) | 453 (66.7) |
| I don’t know | 17 (2.5) | 9 (1.3) |
| **Last time saw dentist** |  |  |
| Never saw a dentist | 561 (82.7) | 518 (76.3) |
| Seen a dentist | 117 (17.3) | 161 (23.7) |
| Within 12 months period | 21 (3.1) | 36 (5.3) |
| >1year but ≤ 2 years | 7 (1.0) | 9 (1.3) |
| > 2years | 89 (13.1) | 116 (17.1) |
| **If yes to having seen a dentist, reason for visit** |  |  |
| Urgent attention | 113 (96.6) | 156 (96.9) |
| Routine check / treatment | 4 (3.4) | 5 (3.1) |
| **Source for the last dental care** |  |  |
| Dentists | 23 (4.2) | 39 (8.2) |
| Other medical practitioners | 85 (15.5) | 31 (6.6) |
| Vendors/ traditional practitioners | 140 (25.6) | 20 (4.2) |
| Patent medicines/ chemist | 247 (45.2) | 287 (28.1) |
| Self-medication | 52 (9.5) | 96 (20.3) |
| **Teeth cleaning aids** |  |  |
| Toothbrush | 531 (78.3) | 644 (94.9) |
| Chewing stick | 114 (16.8) | 17 (2.5) |
| Toothbrush and chewing stick | 27 (4.0) | 18 (2.7) |
| **Use toothpaste to clean the mouth** |  |  |
| Yes | 576 (85.0) | 659 (97.1) |
| Sometimes | 15 (2.2) | 6 (0.9) |
| No | 87 (12.8) | 14 (2.1) |
| **If yes, does tooth paste contains fluoride** |  |  |
| Yes | 436 (73.8) | 532 (80.0) |
| Sometimes | 5 (0.9) | 5 (0.8) |
| No | 10 (1.7) | 8 (1.2) |
| I don’t know | 140 (20.7) | 120 (18.1) |
| **Frequency of mouth cleaning / day** |  |  |
| Less than twice daily | 515 (76.0) | 495 (72.9) |
| Twice daily | 144 (21.2) | 177 (26.1) |
| More than twice daily | 19 (2.8) | 7 (1.0) |
|  |  |  |
