# Supplementary material for: Prevalence and determinants of oral health conditions and treatment needs among slum and non-slum urban residents: Evidence from Nigeria
Source: PLOS Glob Public Health. 2022 Apr 22;2(4):e0000297. doi: 10.1371/journal.pgph.0000297 (PMC10021815; doi:10.1371/journal.pgph.0000297)
Supplement: S2 Table — (DOCX) [file pgph.0000297.s002.docx]

***S2 Table*: *Oral health conditions following dental examination. Number and percentages reported unless stated otherwise.***

| Oral examination outcomes | | Slum  (n=678) | Non-slum  (n=679) | Total  (n=1,357) | p-value |
| --- | --- | --- | --- | --- | --- |
| Caries experience | Dental caries | 181 (26.7) | 154 (22.7) | 335 (24.7) | 0.086 |
|  | Missing teeth | 91 (13.4) | 97 (14.3) | 188 (13.9) |  |
|  | Filled teeth | 15 (2.2) | 7 (1.0) | 22 (1.6) |  |
| Life time caries experience | DMFT* – mean (SD) | 1.1 (2.7) | 1.3 (3.1) | 1.2 (2.9) |  |
| Periodontal disease | Periodontal disease (total) | 522 (77.0) | 374 (55.1) | 896 (66.0) | <0.001 |
|  | Gingival bleeding⁺ | 506 (74.6) | 357 (52.6) | 863 (63.6) | <0.001 |
|  | Periodontal pocket⁺ | 153 (22.6) | 105 (15.5) | 258 (19.0) | 0.001 |
|  | Periodontal attachment loss⁺ | 97 (14.3) | 36 (5.3) | 133 (9.8) | <0.001 |
| Dental erosion | Dental erosion | 67 (9.9) | 44 (6.5) | 111 (8.1) | 0.022 |
| Dental trauma | Dental trauma | 218 (32.2) | 140 (20.6) | 358 (26.3) | <0.001 |
| Oral mucosal lesions | Oral mucosal lesions | 20 (2.9) | 9 (1.3) | 29 (2.1) | 0.039 |
| Enamel fluorosis | Enamel fluorosis | 25 (3.7) | 31 (4.6) | 56 (4.1) | 0.416 |
| Denture use | Denture use | 6 (0.9) | 7 (1.0) | 13 (0.9) |  |
| Level of dental treatment needed (intervention urgency) | No treatment required | 18 (2.7) | 37 (5.4) | 55 (4.1) |  |
|  | Routine treatment required | 420 (61.9) | 453 (66.7) | 873 (64.3) |  |
|  | Prompt treatment required | 194 (28.6) | 148 (21.8) | 342 (25.2) |  |
|  | Urgent treatment needed | 42 (6.2) | 40 (5.9) | 82 (6.0) |  |
|  | Referred** | 4 (0.6) | 1 (0.1) | 5 (0.4) | 0.003 |

* Decayed, missing or filled teeth; Referred**- for comprehensive evaluation or medical/dental treatment (systemic condition); Routine - Required preventive or routine treatment; Prompt- Prompt treatment required (including scaling); Urgent - required immediate (urgent) treatment needed due to pain/ infection of dental & oral origin ⁺ Multiple categories could be selected for the type of periodontal disease (gingival bleeding, periodontal pocket and attachment loss)
