# Supplementary material for: Prevalence and determinants of oral health conditions and treatment needs among slum and non-slum urban residents: Evidence from Nigeria
Source: PLOS Glob Public Health. 2022 Apr 22;2(4):e0000297. doi: 10.1371/journal.pgph.0000297 (PMC10021815; doi:10.1371/journal.pgph.0000297)
Supplement: S3 Table — (DOCX) [file pgph.0000297.s003.docx]

***S3 Table: Logistic regression models to explore associations between dental caries and risk factors, unadjusted and adjusted for age group and sex.***

|  | **Dental caries/Total (%)** | **Unadjusted odds ratio**  **(95% CI)**  **p-value** | **Adjusted odds ratio**  **(95% CI)**  **p-value** |
| --- | --- | --- | --- |
| **Cariogenic diet** | | | |
| Less frequent | 180/724 (25%) | reference | reference |
| More frequent | 155/478 (25%) | 0.98  (0.77 to 1.26)  p=0.873 | 1.02  (0.79 to 1.32)  p=0.882 |
| **Alcohol intake** | | | |
| Didn’t drink alcohol (last 30 days) | 297/1164 (26%) | reference | reference |
| Moderate intake | 26/154 (17%) | 0.59  (0.38 to 0.92)  p=0.020 | 0.66  (0.42 to 1.05)  p=0.082 |
| Excessive intake | 12/39 (31%) | 1.39  (0.65 to 2.59)  p=0.461 | 1.57  (0.76 to 3.24)  p=0.225 |
| **Tobacco use** | | | |
| Never used | 301/1205 (25%) | reference | reference |
| Ever used | 34/152 (22%) | 0.87  (0.58 to 1.30)  p=0.482 | 0.96  (0.63 to 1.46)  p=0.836 |
| **Teeth cleaning frequency** | | | |
| < twice daily | 237/1010 (23%) | reference | reference |
| ≥ twice daily | 98/249 (28%) | 1.28  (0.97 to 1.69)  p=0.075 | 1.28  (0.97 to 1.69)  p=0.079 |

* Adjusted for age group and sex
