# Supplementary material for: Prevalence and determinants of oral health conditions and treatment needs among slum and non-slum urban residents: Evidence from Nigeria
Source: PLOS Glob Public Health. 2022 Apr 22;2(4):e0000297. doi: 10.1371/journal.pgph.0000297 (PMC10021815; doi:10.1371/journal.pgph.0000297)
Supplement: S4 Table — (DOCX) [file pgph.0000297.s004.docx]

***S4 Table: Logistic regression models to explore associations between periodontal pocket formation and risk factors, unadjusted and adjusted for age group and sex.***

|  | **Periodontal pocket formation/Total**  **(%)** | **Unadjusted odds ratio**  **(95% CI)**  **p-value** | **Adjusted odds ratio***  **(95% CI)**  **p-value** |
| --- | --- | --- | --- |
| **Cariogenic diet** | | | |
| Less frequent | 169/724 (23%) | reference | reference |
| More frequent | 89/633 (14%) | 0.54  (0.41 to 0.71)  p<0.001 | 0.58  (0.44 to 0.78)  p<0.001 |
| **Alcohol intake** | | | |
| Didn’t drink alcohol (last 30 days) | 218/1164 (19%) | reference | reference |
| Moderate intake | 28/154 (18%) | 0.96  (0.62 to 1.49)  p=0.870 | 0.94  (0.59 to 1.50)  p=0.802 |
| Excessive intake | 12/39 (31%) | 1.93  (0.96 to 3.87)  p=0.064 | 2.03  (0.97 to 4.22)  p=0.059 |
| **Tobacco use** | | | |
| Never used | 226/1205 (19%) | reference | reference |
| Ever used | 32/152 (21%) | 1.16  (0.76 to 1.75)  p=0.497 | 1.12  (0.72 to 1.74)  p=0.608 |
| **Teeth cleaning frequency** | | | |
| < twice daily | 181/1010 (18%) | reference | reference |
| ≥ twice daily | 77/347 (22%) | 1.31  (0.97 to 1.76)  p=0.081 | 1.28  (0.95 to 1.73)  p=0.109 |

* Adjusted for age group and sex
