# Supplementary material for: Prevalence and determinants of oral health conditions and treatment needs among slum and non-slum urban residents: Evidence from Nigeria
Source: PLOS Glob Public Health. 2022 Apr 22;2(4):e0000297. doi: 10.1371/journal.pgph.0000297 (PMC10021815; doi:10.1371/journal.pgph.0000297)
Supplement: S2 File — (PDF) [file pgph.0000297.s006.pdf]

TELEGRAMS................ TELEPHONE................

OYO STATE: THE PACESETTER

MINISTRY OF HEALTH

DEPARTMENT OF PLANNING, RESEARCH & STATISTICS DIVISION

PRIVATE MAIL BAG NO. 5027, OYO STATE OF NIGERIA

Your Ref. No. ....
All communications should be addressed to the Honorable Commissioner quoting
Our Ref. No.AD 13/479/ 1247

7th May, 2019

The Principal Investigator,
Department of Periodontology and Community Dentistry,
College of Medicine,
University of Ibadan,
Ibadan.

<u>Attention: Osuh Mary</u>

ETHICS APPROVAL FOR THE IMPLEMENTATION
OF YOUR RESEARCH PROPOSAL IN OYO STATE

This is to acknowledge that your Research Proposal titled: "Determinants of Oral Diseases and Oral Health Care Needs in Slums: A Comparative Study with Non-slum Urban Settings in Nigeria." has been reviewed by the Oyo State Ethics Review Committee.

2. The committee has noted your compliance. In the light of this, I am pleased to convey to you the full approval by the committee for the implementation of the Research Proposal in Oyo State, Nigeria.

3. Please note that the National Code for Health Research Ethics requires you to comply with all institutional guidelines, rules and regulations, in line with this, the Committee will monitor closely and follow up the implementation of the research study. However, the Ministry of Health would like to have a copy of the results and conclusions of findings as this will help in policy making in the health sector.

4. Wishing you all the best.

Signature & Date

Dr. Abbas Gbolahan
Director, Planning, Research & Statistics
Secretary, Oyo State, Research Ethics Review Committee
