# Supplementary material for: Prevalence and determinants of oral health conditions and treatment needs among slum and non-slum urban residents: Evidence from Nigeria
Source: PLOS Glob Public Health. 2022 Apr 22;2(4):e0000297. doi: 10.1371/journal.pgph.0000297 (PMC10021815; doi:10.1371/journal.pgph.0000297)
Supplement: S1 File — (PDF) [file pgph.0000297.s005.pdf]

Biomedical and Scientific Research Ethics Committee
Kirby Corner Road
Coventry
CV4 8UW

Tuesday, 23 July 2019

Dr Mary Osuh
WMS
University of Warwick
Coventry
CV4 7AL

Dear Dr Osuh,

Ethical Application Reference: BSREC 37/18-19
Title: Determinants of Oral Diseases and Oral Health Care Needs in Slums: A comparative study with non-slum urban settings in Nigeria

Thank you for submitting your revisions to the Biomedical and Scientific Research Ethics Committee (BSREC) for consideration. We are pleased to advise you that, under the authority delegated to us by the University of Warwick Research Governance and Ethics Committee, **full approval for your project is hereby granted.**

Before conducting your research it is strongly recommended that you complete the on-line Research Integrity training:
www.warwick.ac.uk/ritraining. Support is available from the BSREC Secretary.

In undertaking your study, you are required to comply with the University of Warwick's Research Code of Practice:
https://warwick.ac.uk/services/ris/research_integrity/code_of_practice_and_policies/research_code_of_practice/

You are also required to familiarise yourself with the University of Warwick's Code of Practice for the Investigation of Research Misconduct:
https://warwick.ac.uk/services/ris/research_integrity/research_misconduct/codeofpractice_researchmisconduct/

You must ensure that you are compliant with all necessary data protection regulations:
https://warwick.ac.uk/services/idc

Please ensure that evidence of all necessary local permissions is provided to BSREC prior to commencing your study.

WARWICK
THE UNIVERSITY OF WARWICK

Please also be aware that BSREC grants **ethical approval** for studies. The seeking and obtaining of all other necessary approvals is the responsibility of the investigator.

Any substantial changes to any aspect of the project will require further review by the Committee and the PI is required to notify the Committee as early as possible should they wish to make any such changes. The BSREC Secretary should be notified of any minor amendments to the study.

May I take this opportunity to wish you the very best of luck with this study.

Yours sincerely

pp.

Dr David Ellard
Chair, Biomedical and Scientific Research Ethics Committee
